# Attitude Sensor from Ellipsoid Observations: A Numerical and Experimental Validation †

**DOI:** 10.3390/s20020433

**Published:** 2020-01-13

**Authors:** Dario Modenini, Alfredo Locarini, Marco Zannoni

**Affiliations:** Department of Industrial Engineering, University of Bologna, 47121 Forlì, Italy; alfredo.locarini@unibo.it (A.L.); m.zannoni@unibo.it (M.Z.)

**Keywords:** attitude determination, horizon sensor, ellipsoid

## Abstract

The preliminary design and validation of a novel, high accuracy horizon-sensor for small satellites is presented, which is based on the theory of attitude determination from ellipsoid observations. The concept consists of a multi-head infrared sensor capturing images of the Earth limb. By fitting an ellipse to the imaged limb arcs, and exploiting some analytical results available from projective geometry, a closed form solution for computing the attitude matrix is provided. The algorithm is developed in a dimensionless framework, requiring the knowledge of the shape of the imaged target, but not of its size. As a result, the solution is less sensitive to the limb shift caused by the atmospheric own radiance. To evaluate the performance of the proposed method, a numerical simulator is developed, which generates images captured in low Earth orbit, including also the presence of the atmosphere. In addition, experimental validation is provided due to a dedicated testbed, making use of a miniature infrared camera. Results show that our sensor concept returns rms errors of few hundredths of a degree or less in determining the local nadir direction.

## 1. Introduction

The determination of spacecraft attitude from a set of vector observations is a recurrent problem, which has been extensively studied for many decades. One of the vector directions that can be profitably exploited for the attitude determination of an Earth orbiter is the local nadir. Indeed, horizon or limb sensors have been traditionally used for this purpose in many missions [1,2,3,4], as they offer reliability and relatively low cost. Standard algorithms for limb sensors rely on the knowledge of the horizon height to compute the nadir direction [3]; as a result, their accuracy, especially in low Earth orbit (LEO), is limited by the variability of the atmospheric layer being detected as the surface of the infrared Earth’s spheroid [2]. 

In recent years, horizon sensors received renewed interest in the aerospace community, as they are becoming a convenient choice for attitude sensing in small satellite platforms, due to the availability of MEMS infrared sensors. In particular, thermopiles have been the most popular choice for miniaturized horizon sensors onboard of CubeSats and micro-satellites [5,6,7,8]. The reported implementations often require accurate modeling of the thermopile response, and the knowledge of the size and range to the imaged target, the Earth. Rated errors are quite variable, spanning from few degrees down to 0.1 deg.

In this paper, we present a novel horizon sensor prototype, which leverages on the availability of low-cost, highly miniaturized infrared cameras having increasingly higher image resolution, for enhancing its accuracy. A short version of this paper with preliminary results validated only through simulations was presented at IEEE MetroAero 2019 [9]: experimental tests are presented in this paper, along with new analysis.

The idea underlying our horizon sensor consists of using an infrared camera to capture one (or more) images of the Earth, and to solve the mathematical problem of attitude determination from the imaged ellipse generated by a target ellipsoid. In particular, it will be shown that exploiting the knowledge of the target shape (i.e., the oblateness), not only the local nadir direction (pitch and roll angles) can be retrieved, but in principle also the rotation about nadir (yaw angle), thus constraining the full attitude. If an image of the planet is made available, the first step is to detect the limb points on the image and fit those points to an ellipse. To this end, it is desirable to capture the entire planetary limb within the camera field of view (FoV), however, the feasibility of such an assumption depends on the orbit altitude. Indeed, for a LEO satellite, the Earth disk may cover more than 130°, which cannot fit within a wide camera FoV. We take, nevertheless, the LEO scenario as a reference to develop a horizon sensor concept, showing that very good attitude determination accuracies can be achieved by combining multiple partial views of the same planetary limb.

Note that the task of computing the attitude of a camera with respect to a celestial body is closely related to the optical navigation problem: this last aims at providing an estimate of the relative position between the spacecraft and an imaged planetary target, assuming the attitude known. Here we deal with the opposite situation, i.e., estimating the attitude when the relative position is known. Despite the optical navigation problem has received lots of interest [10,11,12,13], the attitude determination from images of celestial bodies seems to have received fewer attention [14,15]. In particular, the authors of [14,15] provide solutions to the three-axes attitude determination from the joint observation of the center of the (partially) illuminated planet and of the terminator limb. Such observations fix two directions in the camera frame, namely the observer-to-target direction and the target-to-sun direction. Once these are known, the attitude can be reconstructed using any method available for the classic attitude determination problem from vector observations, i.e., the so-called Wahba’s problem [16]. In this work, instead, we aim at computing the attitude from limb fitting only, without knowledge of the terminator and of the Sun position. Furthermore, our method benefits from the detection of the full limb, thus it is best suited for images in the infrared spectrum, while the cited methods benefit from images in the visible spectrum, as they rely also on the detection of the terminator limb.

The theory upon which our horizon sensor measurement principle relies leads to a quite simple solution for estimating the attitude matrix, which closely resembles an orthogonal Procrustes problem [17]. With respect to some earlier results by the authors [18], the method is refined here in that it now provides the attitude without knowing the size of the target, rather only the shape is required, e.g., the polar and equatorial flattening coefficients. Such a feature is especially useful when dealing with planets having atmospheres, whose impact is to produce a limb shift, i.e., an increase in the effective size of the planet as seen by the sensor.

To the knowledge of the authors, the existing horizon sensor implementation closest to the one proposed herein is found in [19]. In that recent work, a monolithic thermopile array is used to capture a single, partial view of Earth limb, which is assumed as a perfect sphere. Then, two different approaches are considered for estimating the attitude from the extracted limb. The first one relates the attitude to the location of the limb within the image plane. This way, the attitude information depends on the knowledge of the nominal location of the limb in the image at the reference attitude, which in turns depends on the size of the Earth and range to the target. Alternatively, the limb can be fitted to a circle, and the orientation inferred by the location of the circle’s center. This second option is in principle insensitive to the range and target size; however, it relies on the assumption of a spherical target and neglects the effects of the perspective transformation. Indeed, the center of the imaged circle/ellipse is not, in general, the projection of the center of the target (this is true only for a perfectly nadir-pointing camera). Therefore, any method that seeks to measure the attitude straight from the location of the imaged circle/ellipse center would suffer from such an error. As a final drawback, a single, partial view of the limb hardly provides a reliable fit of the entire “Earth disk”.

The method proposed herein attempts to overcome all the above limitations, providing an algorithm for attitude determination, which: (a) makes no restrictive assumptions on the shape of the target, which can be a tri-axial ellipsoid; (b) properly accounts for the perspective transformation; and (c) does not depend on the target size and range to the target, thus being less sensitive to the apparent limb shift due to the atmosphere. As a by-product, the algorithm provides estimate also for the angle about nadir, hence constraining the full attitude, by making use of the non-sphericity of the target. However, this estimate is very coarse for an Earth orbiter, due to the very low flattening of our planet. 

The manuscript is organized as follows: first, we recall the mathematical background of pinhole projective transformation and its action onto quadric surfaces. Then, we formulate the problem of attitude determination from an imaged ellipsoid by studying the transformation between an ellipsoid and the resulting imaged ellipse (Section 2). A covariance analysis of the estimate is also provided in Section 2. Sensor architecture is discussed in Section 3, taking as a reference micro- and nano-satellites as hosting platforms. Section 4 presents the simulation environment developed for testing the attitude determination algorithm through synthetically generated images, while the experimental testbed implemented for the validation of a sensor prototype is described in Section 5. Results of the numerical and experimental test campaigns are then provided in Section 6. Finally, a discussion on the main outcomes of the study is found in Section 7.

## 2. Theory of Attitude Determination from Imaged Ellipsoids

The theory of attitude determination from imaged ellipsoids relies on the algebraic representation of a known result from perspective geometry: the apparent contour of a quadric surface imaged by a projective camera is the intersection of the tangent cone to the ellipsoid, and whose vertex is lying on the camera center, with the image plane; this intersection is a conic. 

In particular, under the roto-translation $T=R\left[\begin{array}{cc} I & t_{w} \end{array}\right]$, the quadric *Q* transforms to the conic *C* on the image plane, according to [20]:

(1) $$K^{-1}C^{*}K^{-T}\propto TQ^{*}T^{T}.$$

In Equation (1) *Q* is the matrix:

(2) $$Q=\left[\begin{array}{cccc} 1/a^{2} & 0 & 0 & 0\\ 0 & 1/b^{2} & 0 & 0\\ 0 & 0 & 1/c^{2} & 0\\ 0 & 0 & 0 & -1 \end{array}\right],$$

*a*, *b*, and *c*, being the ellipsoid semi-axes lengths (for Earth *c* = polar radius, and *a* = *b* = equatorial radius; even though the numerical and experimental validations presented in this manuscript take as a reference scenario the Earth spheroid, all the following theory holds for an arbitrary triaxial ellipsoid); *R* is the orthogonal attitude matrix mapping from the world frame, defined by the ellipsoid axes, to the camera frame (the unknown to be estimated); ***t****_w_* is the translation vector from the camera center to the origin of the target ellipsoid expressed in world frame (assumed known); $C^{*}$ is the inverse (or the adjugate) of the conic matrix C computed from the coefficients of the ellipse quadratic equation:

(3) $$\begin{array}{c} Ax^{2}+Bxy+Dy^{2}+Ex+Gy+H=0↔\left[x\;y\;1\right]C\left[\begin{array}{c} x\\ y\\ 1 \end{array}\right]=0,\\ C=\left[\begin{array}{ccc} A & \frac{B}{2} & \frac{E}{2}\\ \frac{B}{2} & D & \frac{G}{2}\\ \frac{E}{2} & \frac{G}{2} & H \end{array}\right],\; \end{array}$$

and *K* is the intrinsic camera matrix, representing the projective transformation. For a pinhole camera with optical axis coincident with body axis *z* and the pixel array plane coincident with body *x − y* plane:

(4) $$K=\left[\begin{array}{ccc} f_{x} & \alpha & p_{x}\\ 0 & f_{y} & p_{y}\\ 0 & 0 & 1 \end{array}\right],$$

where *f_x_* and *f_y_* is the focal vector, *p_x_* and *p_y_* are the coordinates of the principal point, and α is the skew-angle (equal zero for orthogonal *x*-*y* axes). 

Note that *C* matrix represents an ellipse in the image plane in homogeneous coordinates, as such it is invariant to a common scaling of all its elements.

By defining matrix $C=K^{T}CK$, Equation (1) can be rewritten as:

(5) $$C^{*}\propto R\left(Q_{3}^{*}-t_{w}t_{w}^{T}\right)R^{T}=R\left(Q_{3}^{*}-\rho^{2}\nu_{w}\nu_{w}^{T}\right)R^{T},$$

where $C^{*}=K^{-T}C^{*}K^{-1}$, ρ is the range from the camera to the target, and $\nu_{w}=t_{w}/\rho$ is the line-of-sight unit vector. An expression for the projection of the point quadric *Q* into the conic *C* may also be derived, but it is more complicated than Equation (5). It retains, however, a clearer physical interpretation, as it relates the target ellipsoid to the tangent cone centered at the camera [21].

Defining $B^{*}=Q_{3}^{*}-\rho^{2}\nu_{w}\nu_{w}^{T}$, Equation (5) becomes:

(6) $$B^{*}\propto R^{T}C^{*}R.$$

Equation (6) is a modified form of the well-known orthogonal Procrustes problem, and is also known as the hand-eye calibration problem [22]. Now, assume that we do not wish the overall target size to be known, rather only the semi-axes ratios. We define a dimensionless matrix $\tilde{B}{}^{*}$:

(7) $$\tilde{B}{}^{*}=\tilde{Q}{}^{*}-{\tilde{\rho }}^{2}\nu_{w}\nu_{w}^{T},$$

where $\tilde{Q}{}^{*}=\mathrm{diag}\left(\left[1,\frac{b^{2}}{a^{2}},\frac{c^{2}}{a^{2}}\right]\right)$ and $\tilde{\rho }=\rho /a$ is an unknown dimensionless range. Equation (6) is rewritten as:

(8) $$\tilde{B}{}^{*}=\tilde{Q}{}^{*}-{\tilde{\rho }}^{2}\nu_{w}\nu_{w}^{T}\propto R^{T}C^{*}R.$$

This matrix equation has now four unknowns, namely $\tilde{\rho }$ and the three independent components of the attitude matrix. The idea is to split the solution in two steps:

1. Find $\tilde{\rho }$ such that $\tilde{B}{}^{*}$ and $C^{*}$ are orthogonally similar matrices apart from an unknown scaling.
2. Once $\tilde{\rho }$ and thus $\tilde{B}{}^{*}$ are known, compute the attitude matrix as the solution of a modified orthogonal Procrustes problem.

To solve step 1, we recognize that a necessary condition for Equation (6) to hold is that any dimensionless rational function of the eigenvalues for the two matrices $\tilde{B}{}^{*}$ and $C^{*}$ shall be the same. One of such functions is, for example:

(9) $$0<\frac{{\mathrm{tr}}^{2}\left(\tilde{B}{}^{*}\right)}{\mathrm{tr}\left(\tilde{B}{}^{*}{}^{2}\right)}=\frac{{\mathrm{tr}}^{2}\left(C^{*}\right)}{\mathrm{tr}\left(C^{*}{}^{2}\right)}=\frac{{\left(\lambda_{c,1}+\lambda_{c,2}+\lambda_{c,3}\right)}^{2}}{\lambda_{c,1}^{2}+\lambda_{c,2}^{2}+\lambda_{c,3}^{2}}=k_{c},$$

where:

(10) $$\mathrm{tr}\left(\tilde{B}{}^{*}\right)=\mathrm{tr}\left(\tilde{Q}{}^{*}\right)-{\tilde{\rho }}^{2},\;\mathrm{tr}\left(\tilde{B}{}^{*}{}^{2}\right)=\mathrm{tr}\left(\tilde{Q}{}^{*}{}^{2}\right)+{\tilde{\rho }}^{4}-2{\tilde{\rho }}^{2}k_{q},$$

and $k_{q}=\nu_{w}^{T}\tilde{Q}{}^{*}\nu_{w}\leq 1$. Substituting Equation (10) into Equation (9) leads to the following second order equation in $t={\tilde{\rho }}^{2}$:

(11) $$\left(1-k_{c}\right)t^{2}-2\left[\mathrm{tr}\left(\tilde{Q}{}^{*}\right)-k_{q}k_{c}\right]t+\left[{\mathrm{tr}}^{2}\left(\tilde{Q}{}^{*}\right)-k_{c}tr\left(\tilde{Q}{}^{*}{}^{2}\right)\right],$$

which admits analytical solutions. Equation (11) will have two real roots, whether of different signs or both positive. In the former case, the correct root is the positive one. If both roots are positive, then one should check the one which better matches another dimensionless rational function of the eigenvalues of $\tilde{B}{}^{*}$ and $C^{*}$, such as $\frac{{\mathrm{tr}}^{3}\left(\tilde{B}{}^{*}\right)}{\det\left(\tilde{B}{}^{*}\right)}=\frac{{\mathrm{tr}}^{3}\left(C^{*}\right)}{\det\left(C^{*}\right)}$. Knowing ${\tilde{\rho }}^{2}$, matrix $\tilde{B}{}^{*}$ is fully determined. Now, following the method devised in [18], we consider the spectral decompositions of $C^{*}$ and $\tilde{B}{}^{*}$:

(12) $$C^{*}=VD_{C}V^{T};\tilde{B}{}^{*}=WD_{B}W^{T},$$

with *V*, *W* orthogonal matrices. Then, it is easy to verify that by setting

(13) $$R=VW^{T}.$$

Equation (16) is satisfied, provided that the eigenvalues are arranged in the same order relative to each other. Actually, any matrix of the form:

(14) $$R=VSW^{T},$$

with *S* = diag{±1±1±1} being signature matrices, will be a solution, too. In [18], it was proved that Equation (13) provides an optimal estimate of R in a least squares sense. Of the eight possible solutions given by Equation (14) only four will have determinant equal to +1, thus being proper rotation matrices. Of these four, only two corresponds to the camera pointing towards the ellipsoid. As a result, there will be a two-fold ambiguity left in the solution that cannot be resolved, unless some additional independent information is available, e.g., past attitude history or angular information obtained from other sensors.

Note that the rotation matrix computed according to Equation (14) allows, in principle, to constrain the full attitude of the spacecraft when imaging a tri-axial ellipsoid. For a spherical target, the yaw angle (about nadir) is clearly unobservable due to spherical symmetry. When imaging a spheroid (i.e., an ellipsoid of revolution, such as the Earth), the capability of detecting the yaw angle depends on the vantage point: whenever the local vertical is aligned to the axis of revolution, then the symmetry of the target prevents yaw observability [18]. Furthermore, the detectability of the orientation about nadir is also degraded when the difference between the polar and equatorial semi-axes gets smaller: unfortunately, that is the case for a spacecraft horizon sensor targeting the Earth, which features a very low flattening (1/298). Nevertheless, it is interesting to assess whether at least a coarse estimate of the yaw angle can be achieved in such a scenario.

### Covariance Analysis

The value of an estimate is as equally as important as the knowledge of its uncertainty. To provide an estimate of the estimated *R* matrix accuracy, we first aimed at a first-order perturbation analysis of Equation (14) subject to perturbations $\delta C^{*},\delta \tilde{B}{}^{*}.$ The former accounts for error in the best-fitted ellipse to the detected limb points, the latter accounts for errors in the ellipsoid model and in the observation direction. 

The derivatives of eigenvalues and eigenvectors of real symmetric matrices admit quite simple analytical formulations. In particular, for any real symmetric matrix *A*, having eigenvalue–eigenvector couples ($\upsilon_{i},u_{i}$), subject to a perturbation δA, it holds [23,24]:

(15) $$\begin{array}{c} \delta u_{i}={\left(\upsilon_{i}I-A\right)}^{+}\delta Au_{i},\\ \delta \upsilon_{i}=u_{i}{}^{T}\delta Au_{i}, \end{array}$$

where the superscript + denotes the Moore-Penrose pseudoinverse. Equation (15) allows us to compute the variation of the spectral decomposition of $C^{*};\;\tilde{B}{}^{*}$, as a function of some applied perturbations $\delta C^{*},\delta \tilde{B}{}^{*}$. Note that these equations indicate that the eigenvector perturbation lies in the plane orthogonal to the unperturbed eigenvector. Furthermore, they hold only for eigenvalues with multiplicity one, as multiple eigenvalues bifurcate, thus are non-differentiable [23]. The variation in the estimated *R* follows directly by differentiation of Equation (14):

(16) $$\delta R=\delta VSW^{T}+VS\delta W^{T}.$$

When dealing with perturbed matrices for covariance computation, it is convenient to switch to the vectorized matrix representation using the “vec” operator, which stacks the columns of a matrix one underneath the other. To this end, we reshaped the first of Equation (15) in a vectorized form:

(17) $$\begin{array}{c} \mathrm{vec}\left(\delta V\right)=\left[\begin{array}{c} {\left(\lambda_{1}I-C^{*}\right)}^{+}⊗v_{1}{}^{T}\\ {\left(\lambda_{2}I-C^{*}\right)}^{+}⊗v_{2}{}^{T}\\ {\left(\lambda_{3}I-C^{*}\right)}^{+}⊗v_{3}{}^{T} \end{array}\right]\mathrm{vec}\left(\delta C^{*}\right)=M\mathrm{vec}\left(\delta C^{*}\right),\\ \mathrm{vec}\left(\delta W\right)=\left[\begin{array}{c} {\left(\mu_{1}I-B^{*}\right)}^{+}⊗w_{1}{}^{T}\\ {\left(\mu_{2}I-B^{*}\right)}^{+}⊗w_{2}{}^{T}\\ {\left(\mu_{3}I-B^{*}\right)}^{+}⊗w_{3}{}^{T} \end{array}\right]\mathrm{vec}\left(\delta \tilde{B}{}^{*}\right)=N\mathrm{vec}\left(\delta \tilde{B}{}^{*}\right), \end{array}$$

where ⊗ denotes the Kronecker product and ($\lambda_{i},v_{i}$), ($\mu_{i},w_{i}$) are the eigenvalue–eigenvector couples of $C^{*}$, $\tilde{B}{}^{*}$, respectively. It is worth to be noted that, as we deal with 3 × 3 symmetric matrices, their eigen-decomposition admits a simple analytical solution [25].

Vectorization of Equation (16) leads to:

(18) $$\mathrm{vec}\left(\delta R\right)=\left(WS⊗I\right)\mathrm{vec}\left(\delta V\right)+\left(I⊗VS\right)\mathrm{vec}\left(\delta W^{T}\right),$$

where $\mathrm{vec}\left(\delta W^{T}\right)=\mathrm{vec}\left(P\delta W\right)$, P being a permutation matrix. Equation (18) allows computing the desired attitude error covariance matrix defined as $P_{RR}=E\left[\mathrm{vec}\left(\delta R\right)\mathrm{vec}{\left(\delta R\right)}^{T}\right]$, through:

(19) $$\begin{array}{c} P_{RR}=\left(WS⊗I\right)P_{VV}{\left(WS⊗I\right)}^{T}+\left(I⊗VS\right)P_{WW}{\left(I⊗VS\right)}^{T}\\ =\;\left(WS⊗I\right)MP_{CC}M^{T}{\left(WS⊗I\right)}^{T}+\left(I⊗VS\right)PNP_{BB}N^{T}P^{T}{\left(I⊗VS\right)}^{T}, \end{array}$$

starting from the covariance of the errors affecting matrices $C^{*}$ and $B^{*}$:

(20) $$P_{CC}=E\left[\mathrm{vec}\left(\delta C^{*}\right)\mathrm{vec}{\left(\delta C^{*}\right)}^{T}\right];\;P_{BB}=E\left[\mathrm{vec}\left(\delta \tilde{B}{}^{*}\right)\mathrm{vec}{\left(\delta \tilde{B}{}^{*}\right)}^{T}\right].$$

In deriving Equation (19) use has been made of the reasonable assumption of uncorrelated noise sources: $E\left[\mathrm{vec}\left(\delta C^{*}\right)\mathrm{vec}{\left(\delta \tilde{B}{}^{*}\right)}^{T}\right]=0$.

To check the consistency of the analytic covariance formula, we performed a set of Monte Carlo simulations, as follows. We generated 1000 synthetic images of an Earth-like spheroid as captured by a nadir-pointing camera, using Matlab^®^ 3D scene control. All images are created assuming the same relative position and attitude between the camera and the target, and differ one from each other only by some additive random noise. For each image, the limb is detected and fitted to an ellipse. Then, the attitude determination algorithm is run, and the error $\delta C^{*}$ between the true and estimated $C^{*}$ matrices is recorded, along with the estimated attitude error, δR. Once all images are processed, we numerically estimated the covariance matrix $P_{CC}$ from the recorded 1000 samples of $\delta C^{*}$. Having $P_{CC}$ available, we computed for each test case the analytical covariance $P_{RR}$ according to Equation (19) to be compared to the actual estimation errors.

**Figure 1:**
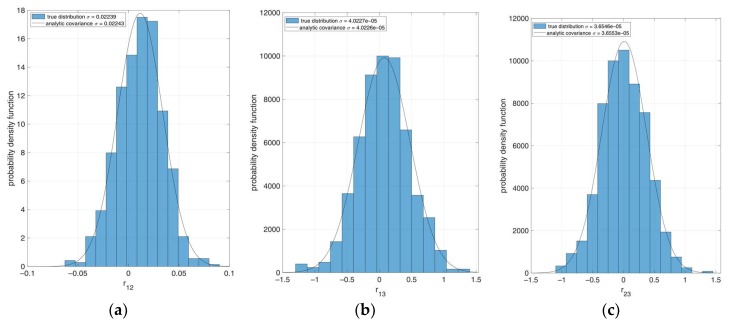
Comparison between the numerically retrieved error distributions and analytic variances for three off-diagonal elements of the attitude matrix. (**a**) *r*_1,2_; (**b**) *r*_1,3_; and (**c**) *r*_2,3_.

The outcome of this process is summarized in Figure 1, where histogram distributions are shown for three off-diagonal elements of the δR matrix, *r_i,j_* (*i* ≠ *j*). Since the assumed nominal *R* matrix is the identity (null attitude) *r_i,j_* is equal, to first order, to the error angle about *k*-axis, so that *r*_1,2_ *r*_1,3_ and *r*_2,3_ correspond approximately to the error angles about the third (yaw), second (pitch)*,* and first (roll) axis, respectively. Superimposed to each histogram, the curve of a Gaussian distribution probability function is shown, having variance equal to the corresponding element in the main diagonal of the analytic $P_{RR}$.

Comparison of the numerical distributions with the theoretical variances shows very good matching. The simulations confirm also that the yaw angle (leftmost panel) was poorly observable, as expected for an imaged spheroid having very low flattening.

## 3. Sensor Architecture

Since computing the attitude from ellipsoid images relies on fitting an ellipse to a set of edge points, it is desirable to have the entire planetary limb lying within the camera field of view. The feasibility of such an assumption depends on the orbit altitude with respect to the planet size and, for LEO, it is hardly to be fulfilled, unless a very wide FoV camera is available, which would induce severe lens distortion. We take, however, this scenario as a target, since a small satellite platform in LEO orbit is deemed to be the most common application for such a kind of attitude sensor.

To cope with a target that exceeds the camera FoV, a multi-head sensor concept was envisaged: here, we required that three simultaneous views of limited limb portions were made available to properly constrain the full “Earth circle” [9]. We therefore considered a sensor assembly formed by a set of three miniaturized infrared camera cores. Two alternative implementations were envisaged, according to the hosting spacecraft platform: configuration (a) for the microsatellite class, and configuration (b) for the nanosatellite class, both being based on commercial, uncooled microbolometer. In particular, a FLIR Boson 320^®^ and a FLIR Lepton^®^ 3 were chosen as the reference for configuration (a) and (b), respectively. Their main characteristics are collected in Table 1 and Table 2 [26,27].

### Measurement Principle: Limb Detection and Fitting

In our sensor conceptual design, the measurement is an ellipse extracted from the image, which consists of five independent scalar parameters. Estimating the ellipse parameters from an image requires two processing steps, namely:

(a) Edge detection;
(b) Edge fitting to an ellipse.

Both these topics are extensively studied in the image processing community, however, since they are not the core of this work, we will not pursue them in detail. 

The multi-head limb sensor demands for an extension of the theory described in Section 2 (originally developed to handle a single ellipsoid view), to fuse the observations from different images into one best-fit ellipse, as follows. Let us first define a body reference frame, with respect to which the orientation of the three sensor heads is specified through matrices $R_{1/b},R_{2/b},R_{3/b}$, respectively. Then, it is easy to verify that, for each image *i*, the following transformed homogeneous limb pixel coordinates ${\tilde{x}}_{i}$:

(21) $${\tilde{x}}_{i}=R_{i/b}^{T}K^{-1}x_{i},$$

are referenced to the common body frame. We then stack the transformed homogeneous coordinates of the limb points detected in the three images into a single vector, ${\tilde{x}}_{st}$:

(22) $${\tilde{x}}_{st}=\left[\begin{array}{c} {\tilde{x}}_{1}\\ {\tilde{x}}_{2}\\ {\tilde{x}}_{3} \end{array}\right].$$

Fitting an ellipse to the stacked vector, the corresponding adjoint matrix will satisfy Equation (6), which provides the desired generalization of the attitude estimation method from multiple partial views of the same ellipsoid. 

It is worth to note that, for the multi-head sensor concept applicability, the three sensor cores do not need to be clustered together in a single mechanical assembly: one just need to know the relative orientation of each camera with respect to the others, and to the common spacecraft reference frame.

To test the consistency of the developed theoretical framework, we set up both a numerical simulation scenario and an experimental testbed, as described in the following sections.

## 4. Numerical Simulator

As a first step towards the validation of the horizon sensor concept, we focused on the testing of the algorithm for attitude determination from multiple views of the limb of an Earth-like target. To this end, we developed a Matlab^®^-based simulation environment, which generates synthetic images of an ellipsoid target with the same flattening of the Earth. The simulator accounts for the main error source affecting the attitude determination from infrared Earth images, namely, the presence of a diffuse, inhomogeneous limb shift, due to the atmosphere. The consequence of a diffuse limb is that of resembling a slightly larger Earth, or a slightly smaller camera distance to the target. A non-homogeneous limb shift, instead, will result to a detected target shape, which differs from the solid Earth ellipsoid. While the former effect is rejected by the proposed algorithm, the latter is expected to induce some errors. The simplified atmospheric model implemented in our simulator aims at assessing, at least approximately, the magnitude of such errors, rather than providing a high-fidelity imaging simulation tool.

**Figure 2:**
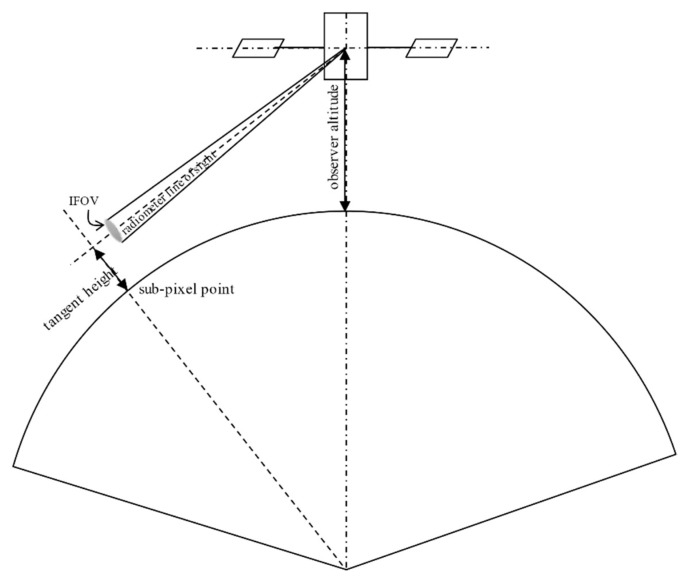
Definition of tangent height for atmosphere modeling.

Several studies are found in the literature, assessing the issue of the stability of our planetary atmosphere over the infrared spectrum, both from a theoretical and experimental standpoint. A widespread descriptor used to characterize the atmospheric stability are the curves of the atmospheric radiance variation as a function of the tangent height, defined as the minimum altitude of the radiometric line of sight, see Figure 2. The main features can be summarized as [2,4]:

- The normalized atmospheric radiance profile decreases with the tangent height roughly following an inverse S-shaped curve.
- The spatial variation of the apparent limb shift has a systematic component, depending mainly on the latitude, plus a stochastic component. The two are almost equally important in magnitude.
- Overall, the variability of the atmospheric radiance induces changes in the detected infrared limb height of about ±10 km.

These features were taken as a guideline to implement a simple model for simulating the effects of a planetary limb on an infrared gathered image, as follows. First, we assumed the atmospheric infrared radiance profile width to be the sum of a nominal, constant value, $\bar{w}$, plus a variable term, dw. The nominal width $\bar{w}$ depends on the band of interest within the IR spectrum; for our study, we assumed $\bar{w}=$ 76 km. The variation dw is modeled as a discrete first-order Markov process function of the latitude λ of each subpixel point:

(23) $$dw\left(\lambda_{k+1}\right)=dw\left(\lambda_{k}\right)e^{-\frac{\Delta \lambda }{\tau }}+u_{k},$$

where the correlation length τ and the standard deviation of the normally distributed random process *u_k_*, were tuned for leading to *dw* variations of up to about ±10 km.

Then, we generated synthetic images through the following steps. Given the assumed relative attitude and position from the camera to the target ellipsoid:

(a) For each pixel *p* of the sensor, compute the nearest point on the target ellipsoid to the line-of-sight vector stemming from the camera centre to the pixel (i.e., the sub-pixel point). This task was accomplished through JPL’s SPICE toolkit routine cspice_npedln.

(b) Compute the distance from that ellipsoid point to the pixel line, i.e., the tangent height, and the latitude of the sub-pixel point.

(c) To each sub-pixel point latitude, an inverse S-shaped atmospheric radiance profile is assigned, having width equal to $\bar{w}+dw$, as predicted by Equation (23); 

(d) The image intensity level at the given pixel, *I(p)*, is assumed to be proportional to the normalized atmospheric radiance profile computed at step (c).

The resulting synthetic images were convoluted using a Gaussian kernel having standard deviation of 1.5 pixels, to resemble the blur effect expected in the infrared spectrum. Then, Gaussian noise was added, and images were finally converted to 16-bit grayscale. 

**Figure 3:**
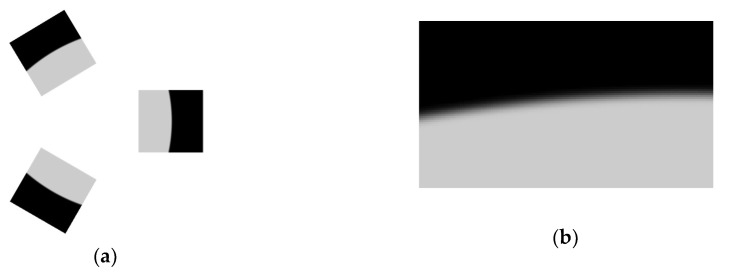
(**a**) Cluster of three simulated limb images and (**b**) detail of the edge region highlighting the blur induced by the atmosphere model [9].

The process above was repeated for generating a cluster of three images (Figure 3, left panel) captured by the three sensor heads. A zoom of the limb region of one of the images is given in Figure 3, right panel, which highlights the variable limb height across the image.

## 5. Experimental Testbed

**Figure 4:**
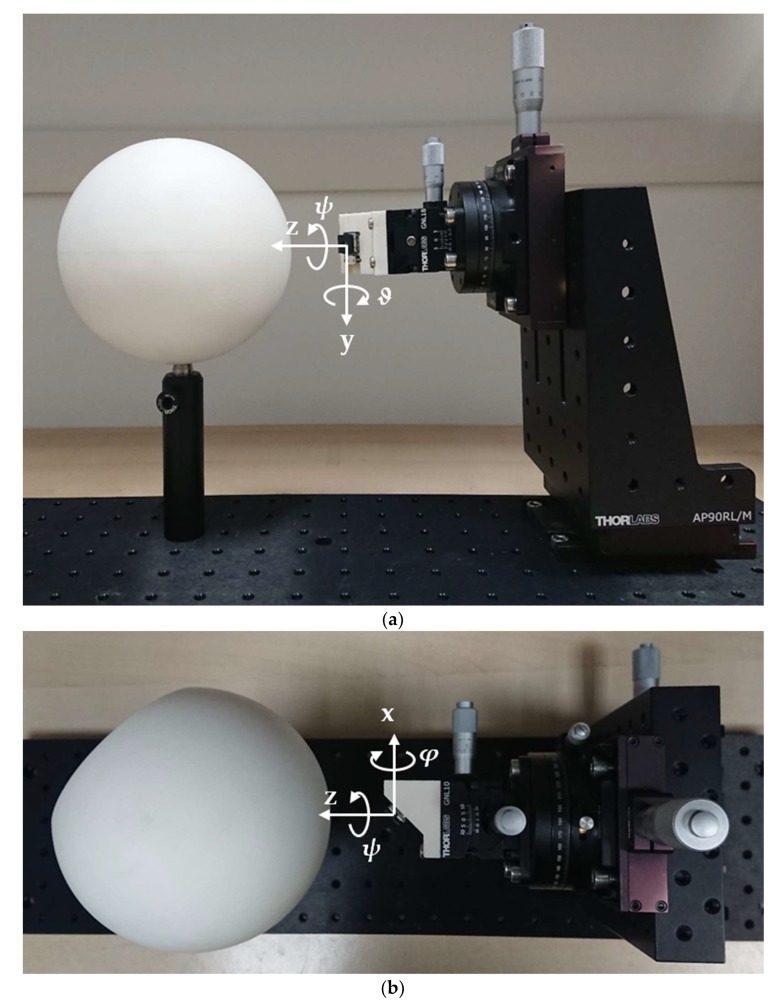
(**a**) Experimental testbed (side-view). (**b**) Experimental testbed (top-view).

The conceptual design of the horizon sensor was verified through a five degrees-of-freedom experimental testbed designed and assembled in our laboratory, making use of opto-mechanics components from Thorlabs Inc. (see Figure 4). The testbed was equipped with one camera and implements a virtual multi-head sensor, which is described in the following. Indeed, using a single camera with variable orientation allowed for a simpler and more cost-effective implementation of the testbed than by using three separated physical cameras, without undermining the algorithm validation. The sensor employed consists of a Flir Lepton^®^ 3 camera (see Table 2), thus representative of a lower resolution sensor implementation targeted to nano-satellites. Three rotational degrees of freedom are provided by micrometric stages, having 10 arcmin of resolution. The target consists of a spheroid made of PVC plastic having nominal radii equal to 68.75 and 69.00 mm, respectively and mounted with the axis of symmetry orthogonal to the test-bed plane. The remaining two translational degrees of freedom allows for fine alignment between the camera and the target center. Rotations are implemented as a 3-1-2 (yaw-roll-pitch) rotation sequence, with angular excursions of 360° about yaw (*ψ*), ±5° and ±10° for the roll (*φ*, inner) and pitch (*ϑ*, outer) axes. Yaw axis is directed towards the spheroid center, pitch axis is orthogonal to the test-bed plane (positive downward), and roll axis directed to create a right-handed triad. The camera itself is then mounted on a PVC support tilted by 45° about pitch axis, for having a portion of the spheroid limb in view at nominal orientation. 

The implemented rotation kinematics allows for the camera to perform a conical scan of the target limb when rotating around the yaw axis. Capturing three successive images at three different camera yaw orientations, allows us to virtually simulate the multi-camera scenario. More precisely, since the conical scan is the first angle of the rotation sequence (performed before the pitch-roll rotations), such configuration is equivalent to prescribe variable camera orientations *R_b/i_* with respect to a fixed body-to-world *R* attitude, rather than assuming a fixed camera-to-body orientation *R_b/i_* w.r.t. a rotating body-to-world attitude, as in an actual operative scenario. However, implementing this last option requires the conical scan to be performed after the pitch-roll rotations, as in a 1-2-3 sequence, which, in turn, is not achievable with the mounting options offered by the three rotational stages in our testbed.

**Figure 5:**
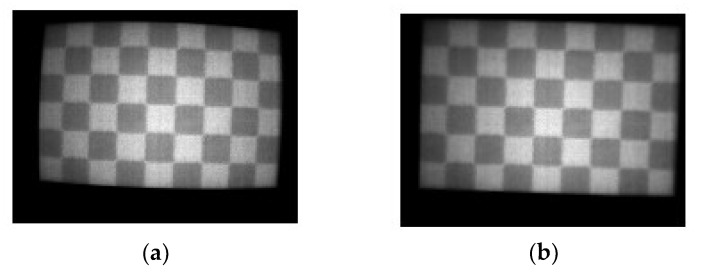
Sample image checkerboard image used for infrared camera calibration. (**a**) Raw and (**b**) undistorted using the estimated radial distortion coefficients.

The theory developed in Section 2 assumes an undistorted pinhole camera model, with known intrinsic matrix *K*; therefore, a calibrated camera is required for the experimental tests. In this work, camera calibration has been performed using Matlab Camera Calibration Toolbox, with a second order radial distortion model. The calibration device consists of a checkerboard pattern printed on an aluminum board (checkerboard size = 15 mm, see Figure 5). To allow for sufficient thermal contrast, the checkerboard is heated up using a heat gun. An index commonly used for evaluating the quality of a camera calibration is the mean reprojection error (MRE) of the corner points’: in this work, an MRE = 0.13 pixels has been achieved after processing 12 checkerboard images. Such MRE value is quite in line with published data on thermal cameras calibration employing standard checkerboard patterns [28], even though lower MRE values can be achieved with dedicated calibration devices [29]. The estimated intrinsic camera parameters are reported in Table 3.

## 6. Results

### 6.1. Results of Numerical Validation

The Matlab tool described in Section 4 was used for numerical validation through extensive simulations, for assessing the expected accuracy of the attitude determination algorithm when capturing images of an Earth-like spheroid with atmosphere, under varying points of observation. In particular, three orbit altitudes were considered, corresponding to different ratios of altitude to Earth radius, namely *h*/*R_E_* = 0.1, 0.2, and 0.3 (note that in the short conference version of this paper [9], the dimensionless orbit altitudes were incorrectly reported as 0.01, 0.02, and 0.03, due to typos). For each altitude, vantage points were generated at latitudes ranging from 0 to 85°, with 5° of angular step. For each vantage point, a set of three images was generated (as if they were captured by the three sensor heads), prescribing a nominally nadir-pointing spacecraft attitude, with some randomly generated, normally distributed off-nadir angles (φ, ϑ having mean and standard deviation *μ* = 0°, *σ* = 1°, respectively). Images resolution corresponds to the one of a micro-satellite targeted camera, as found in Table 1.

**Figure 6:**
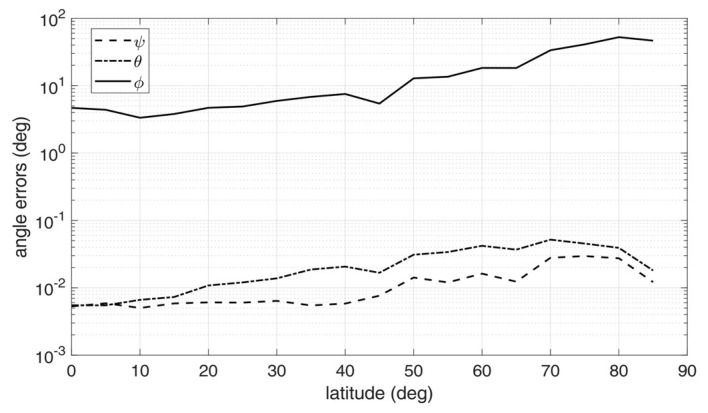
Attitude errors as a function of latitude for an Earth-like spheroid with atmosphere, *h*/*R_E_* = 0.1 [9].

**Figure 7:**
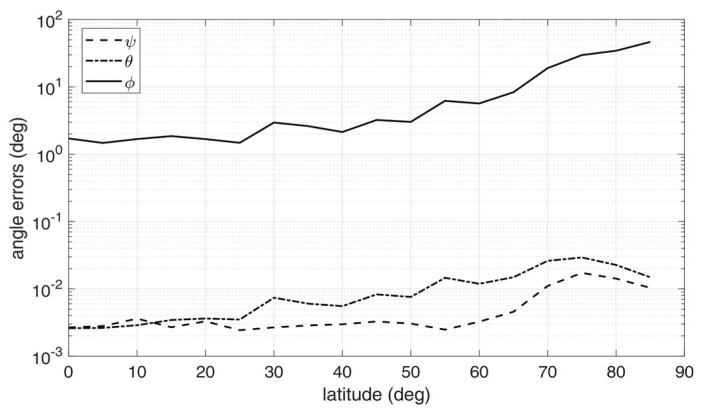
Attitude errors as a function of latitude for an Earth-like spheroid with atmosphere, *h*/*R_E_* = 0.2 [9].

**Figure 8:**
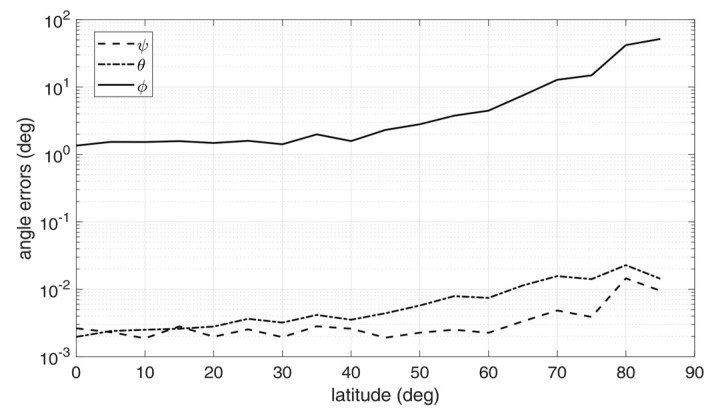
Attitude errors as a function of latitude for an Earth-like spheroid with atmosphere, *h*/*R_E_* = 0.3 [9].

For each triple of images, raw limb points were detected using the method in [30], transformed according to Equation (21), and then fitted to an ellipse using the robust iterative ellipse fitting algorithm of [31]. Then, the attitude determination algorithm was run applying the steps from Equation (11) to (14), and the estimated attitude matrix was compared to the one used to generate the image triple, to compute the attitude error angles. The above procedure was repeated for 30 times, thus providing 30 image triples, and the corresponding attitude errors were root-mean-squared (rms) across the batch. The resulting rms error angles are displayed in Figure 6, Figure 7 and Figure 8, for *h*/*R_E_* = 0.1, 0.2, and 0.3, respectively.

Inspection of the figures suggest that very high accuracy was reached in the estimation of the nadir direction, with rms errors spanning from 10^−3^ to 10^−2^ degrees for pitch and roll angles. On the other hand, the angle about nadir (yaw) could be retrieved only very coarsely, with increasing error as the vantage point approaches the symmetry axis of the Earth spheroid (i.e., for latitudes close to 90°). This is an expected outcome, according to the discussion at the end of Section 2. Overall, the rms yaw error remained below 10° only up to a latitude of 60°. On the other hand, pitch and roll angles accuracies were only marginally degraded when approaching Earth symmetry axis.

Note that the worse accuracy in estimating the yaw angle has a clear physical interpretation, which is well-known also in star tracking sensors [32]: while the off-nadir angles depends mostly on the location of the center of the ellipse in the image, which can be determined with sub-pixel precision, yaw accuracy is related to how well the orientation of the ellipse axes within the image can be determined. If the target resembles a sphere (i.e., it has very low flattening ratios), the imaged ellipse will resemble a circle, and the yaw angle becomes barely detectable: indeed, this is the situation encountered in an Earth sensor scenario.

Finally, performance was also sensitive to orbit altitude: the accuracy tended to improve when increasing the altitude, as a result of the wider portions of the Earth limb being captured by the cameras and, at the same time, of the apparently thinner atmosphere.

### 6.2. Results of Experimental Validation

**Figure 9:**
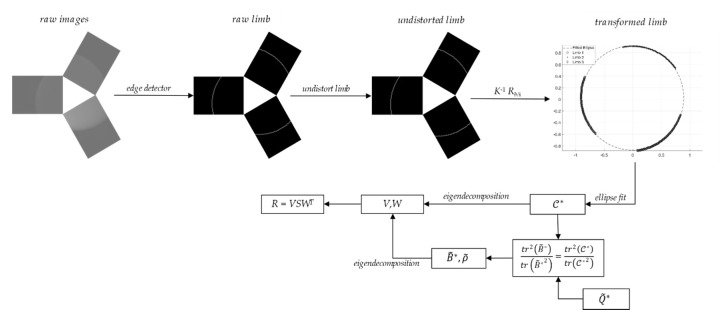
Schematic workflow for the attitude estimation from multiple limb images of an ellipsoid.

Four test cases were considered for the preliminary validation of the sensor prototype, corresponding to different pitch-roll configurations. For all the tests, the camera height was kept aligned to the spheroid center, which corresponds to setting $\nu_{w}={\left[\begin{array}{ccc} 1 & 0 & 0 \end{array}\right]}^{T}$ in Equation (7) when computing $\tilde{B}{}^{*}$ matrix. For each pitch-roll setting, three images were gathered at yaw orientations separated by 120° one from each other. For each image, the limb was extracted using a standard Sobel operator. Limb pixels’ coordinates from raw pictures were undistorted according to the estimated distortion coefficients, using a built-in Matlab function. The undistorted limb coordinates are then transformed according to Equation (21), using the calibrated intrinsic camera parameters in Table 3, and setting matrices *R_b/i_* according to:

(24) $$\begin{array}{c} R_{b/1}=E_{2}\left[45{}^{\circ}-\vartheta \right]E_{1}\left[\varphi \right]E_{3}\left[0{}^{\circ}\right],\\ R_{b/2}=E_{2}\left[45{}^{\circ}-\vartheta \right]E_{1}\left[\varphi \right]E_{3}\left[-120{}^{\circ}\right]\\ R_{b/3}=E_{2}\left[45{}^{\circ}-\vartheta \right]E_{1}\left[\varphi \right]E_{3}\left[120{}^{\circ}\right], \end{array},$$

where *E_j_ j* = 1, 2, 3 denotes the elementary rotation matrix around the *j*-th coordinate axis. Finally, an ellipse was fitted to the stacked vector of transformed points from the three views, using the method in [31]. Having the ellipse matrix available, the attitude determination algorithm was run applying the steps from Equations (11)–(14). The attitude matrix *R* output of this process was compared to the zero-reference attitude matrix $R_{0}$ computed running the algorithm for *φ* = *ϑ* = 0°. We defined the estimation errors as the angles of the 3-1-2 rotation sequence computed from the error attitude matrix $RR_{0}^{T}$. The process was repeated for four test-cases, corresponding to different pitch-roll combinations: the outcome is summarized in Table 4. A schematic representation of the overall measurement workflow, from image capturing to attitude matrix estimation, is given in Figure 9.

By inspection of Table 4, it emerged that the errors in the determination of the off-nadir angles lay in the range 10^−2^ deg, i.e., well below the accuracy limit of the experimental platform. This is evidence that the foreseen measurement procedure leads to results that are consistent with the previous theoretical and numerical analyses, providing high accuracy nadir direction determination. This is a relevant outcome, especially when considering the additional error sources affecting the experimental set-up, such as the imperfect camera distortion compensation, and imperfect knowledge of the relative orientation of the camera when capturing the three images. On the other hand, the estimation of the yaw angle was very poor, and worse than what was predicted by the numerical simulator, which is, nevertheless, not surprising, given the lower image resolution of the FLIR Lepton^®^ 3 camera. 

## 7. Discussion

Some new theoretical results for the attitude determination from multiple limb views of an ellipsoid, together with the increased availability of highly miniaturized infrared cameras, allowed us to design a high accuracy horizon sensor for spacecraft attitude determination in Low Earth Orbit. The sensor makes use of images of the Earth gathered in the infrared spectrum, and on fitting the detected limb to an ellipse. For satellites in LEO, where the target exceeds significantly the camera FoV, a single, partial, limb view is not enough to provide accurate ellipse fitting; therefore, the proposed sensor concept exploits a multi-head implementation, where limb views from three different images are combined for providing a single best-fitted ellipse. The full camera attitude is then estimated starting from the analytic projection of an ellipsoid onto the image plane of a pinhole camera. Once the Earth shape and the direction of observation are known, the camera attitude can be retrieved as the solution to a modified orthogonal Procrustes problem. The proposed solution, being invariant to an unknown scaling of the target size, is less affected by errors due to the atmosphere own radiance. 

The attitude determination algorithm was first checked against synthetically generated images of an Earth-like target, embedding a simple model for a diffuse, inhomogeneous limb to resemble the presence of the atmosphere. Sensitivity of the algorithm accuracy under varying vantage points was assessed through numerical simulations. Obtained results lead to rms errors in the order of 10^−2^ deg (or less) for the off-nadir angles in most operating conditions. The capability of measuring the orientation about nadir was instead very coarse, and strongly dependent on the observation latitude and camera resolution.

The sensor concept was then validated through an experimental testbed, designed to virtually recreate a multi-head sensor, making use of only one low-resolution COTS infrared camera equipped with three rotational DoF. The experiments allowed us to verify the nadir direction estimation down to the resolution of the testbed rotational stages (<10 arcmin). On the other hand, the orientation about nadir can be estimated only very coarsely: its practical usefulness in an operative scenario might be achieved using higher resolution cameras, which remains a topic for further investigations.

Our results look promising when compared to existing static horizon sensors for LEO satellites, and motivated us to further develop the conceptual design presented herein. For example, Selex-Galileo IRES-C and Sodern ST-12 are reported to having 3-sigma errors of 1° and 0.1° respectively [33,34]; Maryland Aerospace MAI-SES has a resolution of 0.25 deg [35]; the thermopile array sensor flown on-board of PSSCT-2 picosatellite from the Aerospace Corporation has a rated error within 0.5° [36].

Future efforts will be mainly devoted to the manufacturing of a complete sensor prototype with higher camera resolution, and to its thorough experimental validation. In parallel, a more comprehensive error budget shall be developed, which accounts for additional error sources, such as the misalignment between the different sensor heads.

## Figures and Tables

**Table 1:** Physical characteristics of the IR camera core, FLIR Boson 320, taken as a baseline for a microsatellite targeted implementation of our horizon sensor prototype.

Image Size (px)	HFoV (deg)	Power (mW)	Weight (g)	Envelope (mm^3^)
320 × 256	50	500 (operating) + 330 (shutter)	20 g	21 × 21 × 30

**Table 2:** Physical characteristics of the IR camera core, FLIR Lepton^®^ 3, taken as a baseline for a nanosatellite targeted implementation of our horizon sensor prototype.

Image Size (px)	HFoV (deg)	Power (mW)	Weight (g)	Envelope (mm^3^)
120 × 160	57	150 (operating) + 500 (shutter)	0.9 g	10.5 × 12.7 × 7.14

**Table 3:** Estimated intrinsic camera parameters after calibration procedure (length unit in pixel).

Focal Lengths *f_x_*, *f_y_*	Principal Point Coordinates	Radial Distortion Coefficients
160.42 ± 1.22	80.70 ± 0.57	−0.333 ± 0.012
160.35 ± 1.22	63.29 ± 0.41	0.212 ± 0.051

**Table 4:** Error angles retrieved during experimental tests.

Test Case	*ψ* Error (°)	*φ* Error (°)	*ϑ* Error (°)
1: *φ* = 5°, *ϑ* = 0°	−3.29	0.03	0.04
2: *φ* = 0°, *ϑ* = 5°	14.20	−0.01	−0.006
3: *φ* = 5°, *ϑ* = 5°	8.05	−0.02	0.02
4: *φ* = 10°, *ϑ* = 5°	−6.88	0.07	−0.001
